# Remote at-home wearable-based gait assessments in Progressive Supranuclear Palsy compared to Parkinson’s Disease

**DOI:** 10.1186/s12883-023-03466-2

**Published:** 2023-12-11

**Authors:** Mansi Sharma, Ram kinker Mishra, Anna J. Hall, Jose Casado, Rylee Cole, Adonay S. Nunes, Gregory Barchard, Ashkan Vaziri, Alexander Pantelyat, Anne-Marie Wills

**Affiliations:** 1 Department of Neurology, Massachusetts General Hospital, Harvard Medical School, Wang ACC Rm 715, 55 Fruit St. , Boston, MA 02114 USA; 2 BioSensics LLC, 57 Chapel St, Suite 200, Newton, MA 02458 USA; 3 Department of Neurology, Johns Hopkins University School of Medicine, 600 North Wolfe Street, Meyer 6-181C, Baltimore, MD 21287 USA

**Keywords:** PSP, Parkinson’s, Wearable sensors, Telemedicine, Remote

## Abstract

**Background:**

Wearable sensors can differentiate Progressive Supranuclear Palsy (PSP) from Parkinson’s Disease (PD) in laboratory settings but have not been tested in remote settings.

**Objectives:**

To compare gait and balance in PSP and PD remotely using wearable-based assessments.

**Methods:**

Participants with probable PSP or probable/clinically established PD with reliable caregivers, still able to ambulate 10 feet unassisted, were recruited, enrolled, and consented remotely and instructed by video conference to operate a study-specific tablet solution (BioDigit Home ™) and to wear three inertial sensors (LEGSys™, BioSensics LLC, Newton, MA USA) while performing the Timed Up and Go, 5 × sit-to-stand, and 2-min walk tests. PSPRS and MDS-UPDRS scores were collected virtually or during routine clinical visits.

**Results:**

Between November, 2021- November, 2022, 27 participants were screened of whom 3 were excluded because of technological difficulties. Eleven PSP and 12 PD participants enrolled, of whom 10 from each group had complete analyzable data. Demographics were well-matched (PSP mean age = 67.6 ± 1.3 years, 40% female; PD mean age = 70.3 ± 1.8 years, 40% female) while disease duration was significantly shorter in PSP (PSP 14 ± 3.5 months vs PD 87.9 ± 16.9 months). Gait parameters showed significant group differences with effect sizes ranging from *d* = 1.0 to 2.27. Gait speed was significantly slower in PSP: 0.45 ± 0.06 m/s vs. 0.79 ± 0.06 m/s in PD (*d* = 1.78, *p* < 0.001).

**Conclusion:**

Our study demonstrates the feasibility of measuring gait in PSP and PD remotely using wearable sensors. The study provides insight into digital biomarkers for both neurodegenerative diseases.

**Trial registration:**

ClinicalTrials.gov Identifier: NCT04753320, first posted Febuary 15, 2021.

**Supplementary Information:**

The online version contains supplementary material available at 10.1186/s12883-023-03466-2.

## Background

Digital health technologies have the potential to provide objective, quantitative digital biomarkers of disease remotely in participants’ homes (reviewed in [1]). While remote in-home wearable sensors have been successfully used to measure PD tremor and motor activity [2–4], such technology has not yet been tested in people with PSP. Remote assessment of disease severity and progression provides several advantages over laboratory or in-clinic evaluations: 1) it allows more frequent, even continuous measurement of the disease; 2) it captures movement in the natural living environment; 3) it significantly reduces the burden of travel for patients with disabilities and for patients who live far from clinical centers [5]. Reducing travel burden is particularly important for people with PSP and their caregivers. These advantages are also relevant for clinical trials, where more frequent remote monitoring and reduced variance in objective continuous measures have the potential to reduce the required sample size.

Gait abnormalities, postural instability, and frequent falls are hallmarks of PSP and falls have been shown to be the most important predictor of survival in this disease [6, 7]. Despite this importance, there are currently no agreed-upon quantitative measures for evaluating gait and balance in PSP (reviewed in [8]). The gait disorder in PSP is quite different from PD and has been described as stiff, clumsy, and lurching “like a drunken sailor” [9]. Gait abnormalities which are highly specific to PSP include the “Rocket sign”, the “I-Beam sign,” (reviewed in [10]) and uncontrolled turning and sitting down, which we have named the “spiral sign.”

Wearable sensors have been shown to differentiate PSP from PD in laboratory settings in several previous studies [11–13]. Gait speed, cadence, and stride length have been previously shown to be significantly reduced in people with PSP compared to idiopathic PD [11–13], even as gait speed and stride length are reduced in PD compared to healthy controls [14]. Sensor-derived measures such as turn velocity, stride length and toe off angle have recently been shown to correlate with the PSP rating scale (PSPRS) and the Movement Disorder Society-Sponsored Revision of the Unified Parkinson's Disease Rating Scale (MDS-UPDRS) [15, 16]. In our study, we hypothesized that it would be possible to perform instrumented measures of gait and balance remotely in the home setting of people with PSP and PD.

## Methods

### Study design

Participants were enrolled in an investigator-initiated observational cohort study performed at two academic medical centers, Massachusetts General Hospital and Johns Hopkins Medical Center. While participants with PSP are being followed for 12 months (at the time of writing), the cross-sectional analysis of the baseline data is presented here. All participants provided written informed consent prior to participation in the study. The protocol and consent forms were approved by the Mass General Brigham Human Research Committee (protocol# 2021P000431) and the Johns Hopkins Medicine Institutional Review Board.

### Study population

The full Exclusion and Inclusion criteria are shown in supplementary Table 1. In brief, participants with probable PSP (by MDS 2017 criteria [17]) or PD (by MDS 2015 criteria [18]) with a caregiver able to assist with all study-related procedures were recruited at Massachusetts General Hospital and Johns Hopkins from November 2021- November 2022. Importantly, enrolled participants were required to ambulate 10 feet unassisted (without the use of a cane or walker) and did not report a history of 5 or more falls per month. These inclusion criteria permitted the safe measurement of gait without any walking aids in the home. If participants were taking dopaminergic medications, motor tasks were performed in their ON state.

### Study procedures

**Fig. 1:**
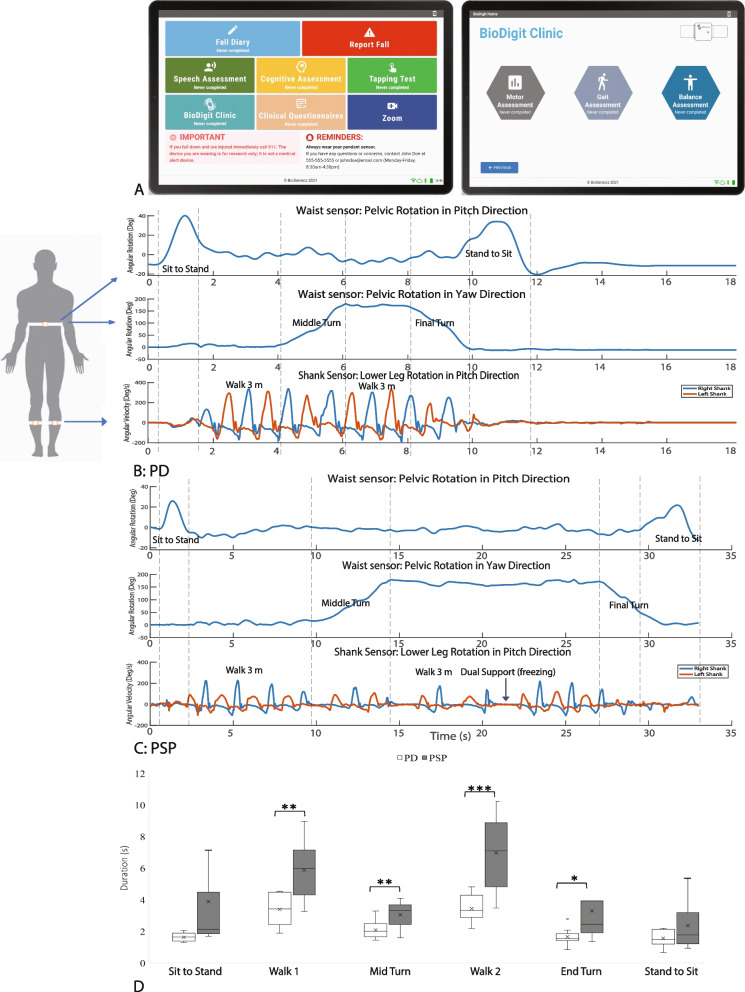
Gyroscope derived data from the LegSys sensors: **A** Shows screenshots of the user interface from the BioDigit Home™ tablet; **B** Shows the gyroscopic data derived from a PD participant wearing the LegSys sensors while performing the Timed Up and Go (TUG) test. **C** Shows the same data derived from a participant with PSP. Note the difference in the scale of the X axis (time in seconds). The top line from each graph shows the waist angular velocity (in degrees); the middle shows the pelvic rotation (in degrees, demonstrating turning); the third line shows the angular position (degrees) of the sensors worn on the shanks. **D** Shows box and whisker plots of the time intervals for each of the components of the TUG test, PD in white, PSP in gray. The box plots show the median (middle line), 25^th^ and 75.^th^ percentiles, and the mean (X). * represents *p* value < 0.05, ** represents *p* value < 0.01, *** represents *p* value < 0.001

A customized system consisting of 3 wearable sensors and a computerized tablet were mailed from the study sites to participants (Fig. 1A). A study coordinator then trained the participants and their caregivers to don the wearable sensors and to perform the digital tasks on the study tablet. The training was provided either virtually over video conference (21 participants) or in the clinic if participants were at the hospital for routine clinical visits (2 participants). Study personnel supervised all activities at home during video conferences (Zoom, San Jose, CA). The presence of a caregiver to assist the participant with tasks and to prevent falls was mandatory. Participants were instructed to skip any assessment deemed unsafe by the trained coordinator or clinician. The study-related assessments involved performing a 2-min walk test (MWT), unobstructed at normal walking speed, a 3-m Timed Up and Go Task (TUG), and 5 times sit-to-stand test. Participants received at least 30 s of rest in between each assessment. Research staff supervised all motor assessments and provided a 3-m ribbon for participants to measure their gait distance for both the 3-m and 2-min walk tests. For the 2-min walk test, participants were instructed to walk back and forth along the longest straight area available (usually the living room) and then to measure the distance of 1 lap. For the TUG test, participants were advised to use a heavy chair with arms positioned to prevent the chair from tipping backward, as described in the recommendations for virtual assessments of the PSPRS [19]. Caregivers were instructed to walk with the participants and to guard against falls during these assessments (Supplementary video 1). The PSPRS, MDS-UPDRS, and Montreal Cognitive Assessment (MoCA) were also collected either virtually (using modified versions of the rating scales [20, 21]) or during in-person visits if participants were evaluated in the clinic. The maximum possible score of the modified virtual 21- itemPSPRS is 72 (removing rigidity, dystonia and ocular motor testing), while the maximum possible score of the modified virtual MDS-UPDRS is 108. Study data were collected and managed using REDCap electronic data capture tools hosted at Johns Hopkins.

### Digital health technologies and measures

Each participant received a Samsung Galaxy Tablet S5e 10.5 running the proprietary application BioDigit Home (BioSensics LLC, Newton, MA, USA). BioDigit Home was custom-designed for this study to include a fall diary, electronic patient-reported outcomes including the Progressive Supranuclear Palsy Quality of Life (PSP-QoL) [22] and the Cortical Basal ganglia Functional Scale (CBFS) [23], speech assessments, and PSP-specific cognitive tests, which will be reported in a separate publication. The BioDigit Home also provides instructional videos for motor assessments (i.e., 2MWT, TUG, and 5 times Sit-to-Stand tests) to ensure homogeneity of administration.

During motor assessments, participants wore 3 LEGSys™ sensors (1 on the anterior shin of each leg and 1 on the lower back, Fig. 1A). All sensors and tablets were provided by BioSensics LLC, Newton, MA, USA. Only 3 sensors were chosen to reduce the complexity of the set-up for participants. The LEGSys sensor consists of a tri-axial accelerometer, gyroscope, and magnetometer sensors, which record movement signals at a sampling frequency of 100 Hz. Spatial–temporal gait parameters, gait variability, and gait phases were calculated using validated algorithms [24]. Angular position was calculated based on the integration of the gyroscope's angular velocity data. Durations of the different components of the TUG (i.e., walk, turn, and sit-to-stand transitions) were quantified using the wearable sensors. Durations of 5 times sit-to-stand repetitions, multi-directional acceleration, and velocity were estimated using the wearable senor attached to the waist. Data captured by the tablet and the wearables were encrypted and securely stored on the tablet and transferred to the HIPAA-compliant BioDigit Cloud server using 4G cellular network technology.

### Statistical analysis

Continuous data were reported as mean ± standard error and categorical data as percentages. Unpaired t-tests, Mann–Whitney U-tests, and chi-square tests were used for comparison according to the scale of the investigated variable and the data distribution. Independent t-tests were used to assess the group effect. The normality and homogeneity of variances were assessed using the Shapiro–Wilk and Levene’s tests (significance threshold *p* > 0.05). The effect size was expressed using Cohen’s d. Values ranging from 0.20 to 0.49 indicate small effects, values between 0.50 and 0.79 indicate medium effects, values ranging from 0.80 to 1.29 indicate large effects, and values above 1.30 indicate very large effects [25]. Furthermore, Spearman correlation analysis was performed to quantify the association between the ordinal (i.e., clinical outcomes) and continuous variables (i.e., sensor-derived measures). All statistical analyses were performed using SPSS statistical software version 28 (IBM). Given the multiple tests performed, we considered a *p*-value less than 0.001 as significant to reject the null hypothesis.

## Results

**Fig. 2:**
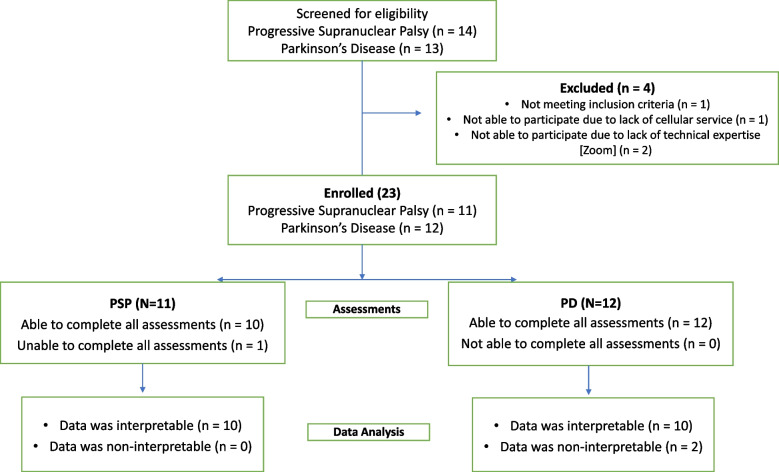
Flow chart of the disposition of all participants in the study

Between November, 2021 and November, 2022, a total of 27 participants were consented and screened of whom 4 were excluded including 2 who had technical difficulties managing the video conferencing software and 1 who did not have cellular service in their area (Fig. 2, Flow Diagram). Of the 23 participants who were enrolled, 11 were diagnosed with PSP (all of whom had the Richardson syndrome variant of PSP) and 12 with clinically established PD. All but 1 PSP participant were able to complete all of the assessments. Of the PD participants, 2 participants’ data was uninterpretable and excluded due to poor placement of the sensors or shifting of the sensors during the assessments resulting in noticeable motion artifacts. In the end, data from 10 PSP and 10 PD participants were included in our analysis. Table 1 shows the baseline demographic and clinical characteristics. No significant differences were observed in age, sex, ethnicity or years of education. Cognition was slightly lower in PSP participants (MoCA score was 23.1 ± 1.5 in PSP vs 26.5 ± 0.6 in PD, *p* = 0.03) and PSP participants had a higher mean Hoehn & Yahr stage despite our attempts to match disease severity by enrolling PD participants with much longer disease duration (91.7 ± 16.2 months since diagnosis in PD, vs 16.9 ± 4.8 months in PSP, *p* = 0.007). Importantly, no adverse events (including falls) occurred during the assessments and caregivers were able to manage the technology while also supervising the participants.

**Table 1:** Participants demographics and clinical characteristics (Mean ± Standard Error)

	PD (*n* = 10)	PSP (*n* = 10)	*p*-value	Cohen’s d
**Demographics**				
Age, *years*	70.3 ± 1.8	67.6 ± 1.3	0.25	0.53
Sex, Female (%)	40	40	1	
Education, *years*	18.0 ± 0.8	17.6 ± 0.8	0.88	0.07
Caucasian, %	70	80	0.6	
**Clinical characteristics**				
Time since diagnosis, *months*	91.7 ± 16.2	16.9 ± 4.8	**0.007**	1.37
Time since symptom onset, *months*	111.3 ± 18.3	50.7 ± 7.5	**< 0.001**	1.98
Cognitive function (MoCA), score	26.5 ± 0.6	23.1 ± 1.5	**0.03**	1.03
MDS-UPDRS part III (video)	33.9 ± 11.7			
Hoehn and Yahr, *score*	2.4 ± 0.18	3.3 ± 0.67	**0.007**	1.41
mPSPRS-21 score (video)		23.1 ± 5.9		

*PD* Parkinson’s Disease, *PSP* Progressive supranuclear palsy, *MDS-UPDRS* Movement Disorders Society Unified Parkinson’s Disease Rating Scale (without rigidity and postural stability20), *mPSPRS-21* Modified virtual PSP Rating Scale; Outcomes with *p* values < 0.05 were considered significantly different and are shown in bold

Figure 1 demonstrates the gyroscopic data from the LEGSys sensors from a participant with PD (Fig. 1B) compared to PSP (Fig. 1C) performing the TUG test. Figure 1D and Supplementary Table 2 summarize the group comparison on the mobility metrics estimated while performing the TUG test between groups. PSP participants were slower performing every element of the TUG, many of which were significant. The average duration for completing the TUG for participants with PSP was 25.49 s compared to 13.8 s in PD participants (*p* = 0.002, *d* = 1.6, note the difference in scale in Fig. 1B and C). Similarly, the time to complete turning in the middle of the TUG test (*p* = 0.009, *d* = 1.37), the first 3 m walk (*p* = 0.001, *d* = 1.67), and the second 3 m walk (*p* < 0.001, *d* = 2.04) were all higher for the participants with PSP compared to PD participants (Fig. 1D). While sit-to-stand transitions were on average slower in PSP as well, these were not significant due to the high degree of variance. Unexpectedly, pelvic sway appeared to be reduced in PSP compared to PD. Figure 1C also demonstrates the high degree of freezing during mid-turns and shows the PSP patient sitting down in the middle of their final turn in an uncontrolled manner, which we are dubbing the “spiral” sign (also shown in the supplementary video).

**Fig. 3:**
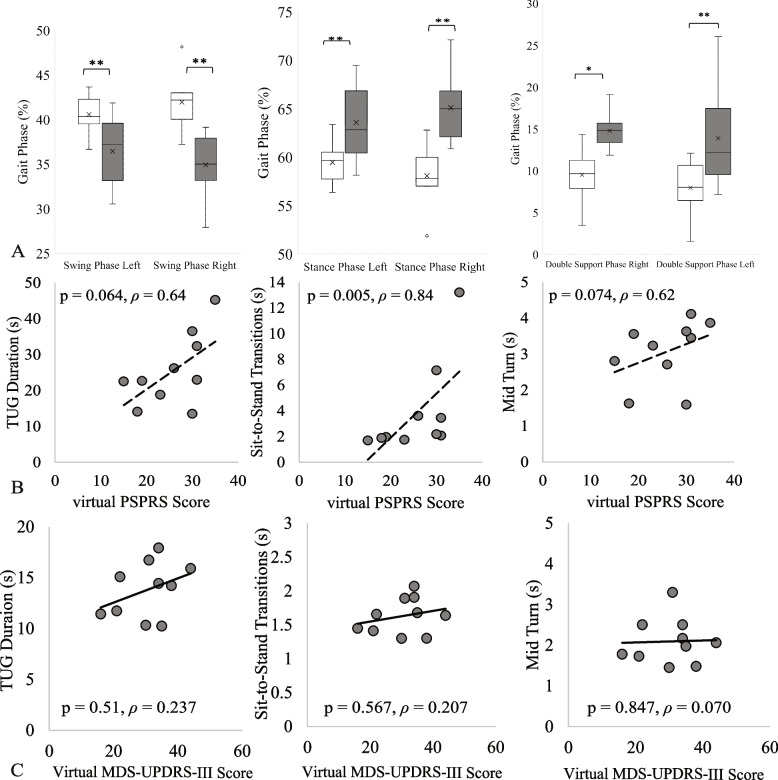
The top row shows box and whisker plots comparing the gait parameters estimates from the 2-min walk test for PD in white, PSP in gray. The box plots show the median (middle line), 25^th^ and 75^th^ percentiles, and the mean (X). The second row shows the Spearman correlations *p* between components of the TUG test and the modified PSPRS. **C** Shows the Spearman correlations *p* between components of the TUG test and the virtual UPDRS part III. * represents *p* value < 0.05, ** represents *p* value < 0.01, *** represents *p* value < 0.001

Table 2 and Fig. 3A summarize the comparison of the gait parameters estimated while performing the 2-min walk test between groups. Gait speed, double support phase estimated during walking, stance phase and swing phase were all significantly different in PSP compared to PD. The double stance phase, which is prolonged in gait freezing*,* constituted 28.7% of the gait cycle for participants with PSP against 17.5% observed for the participants with PD (*p* < 0.001, *d* = 2.19).

**Table 2:** Gait parameters obtained from 2-min walk test (Mean ± Standard Error)

	PD (*n* = 10)	PSP (*n* = 10)	*p*-value	Cohen’s d
**Spatial–Temporal Gait Parameters**				
Gait Speed, *m/s*	0.79 ± 0.06	0.45 ± 0.06	** < 0.001**	1.78
Stride Length, *m*	0.93 ± 0.08	0.59 ± 0.07	0.002	1.65
Stride Time, *s*	1.2 ± 0.03	1.4 ± 0.10	0.12	0.74
Cadence, *steps/minutes*	99.5 ± 2.8	90.2 ± 6.6	0.21	0.58
Total Distance, *m*	89.6 ± 7.5	49.5 ± 7.3	0.01	1.71
**Gait Variability**				
Gait Speed Variability, *m/s*	0.17 ± 0.02	0.17 ± 0.02	0.37	0.45
Stride Time Variability, *s*	0.15 ± 0.03	0.29 ± 0.05	0.13	0.77
Stride Length Variability, *m*	0.17 ± 0.02	0.20 ± 0.02	0.048	1.04
**Phases of Gait**				
Total Double Support Phase, *%*	17.5 ± 1.6	28.7 ± 1.8	** < 0.001**	2.19
Double Support Phase Left, *%*	8.0 ± 1.0	13.9 ± 1.8	0.012	1.30
Double Support Phase Right, *%*	9.5 ± 1.0	14.7 ± 0.6	** < 0.001**	1.97
Stance Phase Left, *%*	59.4 ± 0.6	63.6 ± 1.2	0.006	1.40
Stance Phase Right, *%*	58.0 ± 0.9	65.1 ± 1.0	** < 0.001**	2.27
Swing Phase Left, *%*	40.6 ± 0.6	36.4 ± 1.2	0.006	1.39
Swing Phase Right, *%*	41.9 ± 0.9	34.9 ± 1.0	** < 0.001**	2.27

*m* represents distance in meters, *s* represents time in seconds; gait parameters with *p* values < 0.001 were considered significantly different and are shown in bold; *PD* Parkinson’s Disease and *PSP* Progressive supranuclear palsy

Supplementary Table 3 compare the results of the 5 times sit-to-stand test. One participant with PSP could not finish the 5 times Sit-to-Stand test due to loss of balance. The total duration was 18.0 ± 1.1 s in PD vs 29.5 ± 6.0 s in PSP (*p* = 0.062, *d* = 0.92). While participants with PSP took longer to perform sit-to-stand transitions (*p* = 0.031, *d* = 1.12) with higher variability (*p* = 0.049, *d* = 1.01), these did not reach our pre-specified level of significance.

As shown in Fig. 3B and Supplementary Table 4, several sensor-derived measures from the TUG test, including Sit-to-Stand Transition, total TUG duration, and mid-turns correlated with the modified virtual PSPRS with Spearman correlation coefficients from 0.64–0.84. The duration of Sit-to-Stand transitions correlated with the total modified virtual PSPRS score with a *ῥ* = 0.84, *p* = 0.005 although this did not reach our prespecified level of significance. Correlations between gait measures and part III of the virtual UPDRS were not significant (Fig. 3C).

## Discussion

In summary, we found that remote monitoring in participants’ homes using digital health technologies including wearable devices is safe, feasible, and able to objectively quantify gait and balance measures in PSP and PD. 85% of participants were able to complete these virtual assessments with caregiver support and over 90% of the data generated in participants’ homes were interpretable. We found that using wearable sensors in a natural setting (at home) enabled collection of objective continuous measures and that gait speed and different phases of gait were significantly different in PSP compared to PD. Remote sensors may have clinical value in cases of diagnostic uncertainty, and could supplement or substitute for the items of the PSPRS that are poorly assessed virtually [20].

There are multiple caveats to our study. First, the sample size was small, which led to inadequate power to detect significant differences in many of the variables tested. Second, participants were not representative of the entire spectrum of the disease, based on their ability to ambulate, their MoCA scores, and the fact that we enrolled only people with PSP- Richardson syndrome (PSP-RS). Despite the relatively early stage of PSP participants included in this study, their H&Y stage was significantly more advanced than for PD participants, despite a significantly longer disease duration in PD. Indeed, this was the case despite our deliberate recruitment of PD participants with more advanced disease and impaired gait, underscoring the early gait and balance impairments in PSP-RS. This imbalance underscores the difficulty in matching participants with these diseases based on severity. Third, virtual clinical assessments and virtual rating scales were used, which limited our ability to measure rigidity, dystonia and postural stability. This may in part explain why the correlations between the sensor data and the clinical scales were not as strong as those published by other groups who used in-person laboratory assessments. However, our data show that these clinical assessments are not necessary to differentiate the two diseases.

We are currently following all enrolled PSP participants remotely to measure disease progression over 12 months. We predict that sensor-derived outcome measures will be more sensitive to disease progression and have less variance than the ordinal and rater-dependent PSPRS. We anticipate that this will allow future clinical trials in PSP to enroll smaller participant numbers and to include remote assessments that can improve access to research and decrease participant burden.

### Supplementary Information

**Additional file 1: Supplementary Table 1. **Full inclusion and exclusion criteria. **Supplementary Table 2.** Mobility metrics obtained from Timed-up-Go test (TUG) (Mean ± Standard Error). **Supplementary Table 3**. Five times Sit-to-Stand Test (Mean ± Standard Error). **Supplementary Table 4.** Correlation of TUG metrics with PSPR-Gait and mPSPRS-21 Scores.

**Additional file 2: Supplementary Video.** Participant and her caregiver are shown wearing the 3 sensors at home and performing the Timed Up and Go test. Note the close supervision by her caregiver and the participant’s difficulty sitting down (which we have dubbed the “Spiral sign”).

## Data Availability

The data that support the findings of this study are available only for non-commercial use from the corresponding author, upon reasonable request.
