# Supplementary material for: Remote at-home wearable-based gait assessments in Progressive Supranuclear Palsy compared to Parkinson’s Disease
Source: BMC Neurol. 2023 Dec 11;23:434. doi: 10.1186/s12883-023-03466-2 (PMC10712191; doi:10.1186/s12883-023-03466-2)
Supplement: Supplementary file 1 — Additional file 1: Supplementary Table 1. Full inclusion and exclusion criteria. Supplementary Table 2. Mobility metrics obtained from Timed-up-Go test (TUG) (Mean ± Standard Error). Supplementary Table 3. Five times Sit-to-Stand Test (Mean ± Standard Error). Supplementary Table 4. Correlation of TUG metrics with PSPR-Gait and mPSPRS-21 Scores. [file 12883_2023_3466_MOESM1_ESM.docx]

| **Supplementary Table 1.** Full inclusion and exclusion criteria | |
| --- | --- |
| **Inclusion Criteria** | - Clinical diagnosis of possible or probable PSP phenotype as defined by the 2017 MDS criteria or clinical diagnosis of at least probable PD as defined by the 2015 MDS criteria. - Male or female, aged 18 years to 89 years, inclusive. - Fluent in reading and speaking English. - Capable of providing informed consent based on the investigator's judgment. - Able to comply with the protocol based on the investigator's judgment. - Able to walk 10 feet unassisted - With access to a caregiver who is able to assist with all study-related procedures. |
| **Exclusion Criteria** | - Any neurological, medical, or psychiatric condition that would preclude participation in study activities based on the investigator's judgment. - A history of frequent falls defined as more than 5 falls/month or requirement of a walker to ambulate safely. |

| **Supplementary Table 2. Mobility metrics obtained from Timed-up-Go test (TUG) (Mean ± Standard Error)** | | | | |
| --- | --- | --- | --- | --- |
|  | PD  (n = 10) | PSP  (n = 10) | p-value | Cohen’s d |
| TUG Duration, *s* | 13.80 ± 0.86 | 25.49 ± 3.16 | 0.002 | 1.6 |
| Mid Turn Duration, *s* | 2.09 ± 0.18 | 3.06 ± 0.28 | 0.009 | 1.37 |
| Last Turn Duration, *s* | 1.66 ± 0.16 | 3.31 ± 0.73 | 0.043 | 0.98 |
| Sit-to-Stand Duration, *s* | 1.63 ± 0.08 | 3.90 ± 1.16 | 0.067 | 0.87 |
| Stand-to-Sit Duration, *s* | 1.57 ± 0.16 | 2.38 ± 0.49 | 0.136 | 0.70 |
| Walk Towards, s | 3.40 ± 0.32 | 5.87 ± 0.57 | **0.001** | **1.67** |
| Walk Return, s | 3.45 ± 0.26 | 6.97 ± 0.73 | **<0.001** | **2.04** |
| AP sway during Turning, *deg* | 6.1 ± 0.59 | 5.7 ± 0.57 | 0.64 | 0.21 |
| ML sway during Turning, *deg* | 4.6 ± 0.31 | 3.9 ± 0.41 | 0.19 | 0.61 |
| Pelvis sway velocity during Turning, *deg/s* | 36.1 ± 2.89 | 24.1 ± 2.37 | 0.005 | 1.44 |
| *s* represents time in seconds; *AP* represents anterior-posterior, *ML* represents medial-lateral; mobility metrics with *p values <0.001* were considered significantly different; PD, Parkinson’s Disease and PSP, Progressive supranuclear palsy | | | | |

| **Supplementary Table 3. Five times Sit-to-Stand Test (Mean ± Standard Error)** | | | | |
| --- | --- | --- | --- | --- |
|  | PD  (n = 10) | PSP  (n = 9) | p-value | Cohen’s d |
| Total duration, *s* | 18.0 ± 1.1 | 29.5 ± 6.0 | 0.062 | 0.92 |
| Sit-to-Stand Duration, *s* | 1.56 ± 0.08 | 2.19 ± 0.27 | 0.031 | 1.12 |
| Stand-to-Sit Duration, *s* | 1.82 ± 0.12 | 2.43 ± 0.28 | 0.051 | 1.00 |
| Sit-to-Stand Duration Variability, *s* | 0.13 ± 0.03 | 0.60 ± 0.24 | 0.049 | 1.01 |
| Stand-to-Sit Duration Variability, *s* | 0.22 ± 0.03 | 0.55 ± 0.26 | 0.170 | 0.68 |
| Angular Acceleration Sit-to-Stand, *deg/s^2^* | 203.9 ± 31.9 | 141.3 ± 23.5 | 0.14 | 0.71 |
| Angular Velocity Sit-to-Stand, *deg/s* | 31.9 ± 3.3 | 26.7 ± 3.2 | 0.283 | 0.51 |
| Angular Acceleration Stand-to-Sit, *deg/s^2^* | 182.9 ± 26.5 | 139.2 ± 29.5 | 0.280 | 0.51 |
| Angular Velocity Stand-to-Sit, *deg/s* | 28.6 ± 3.4 | 25.1 ± 4.0 | 0.52 | 0.30 |
| CoV Angular Acceleration Sit-to-Stand, *%* | 22.4 ± 3.6 | 44.9 ± 12.2 | 0.08 | 0.86 |
| CoV Angular Velocity Sit-to-Stand, *%* | 15.8 ± 3.8 | 25.8 ± 7.9 | 0.258 | 0.537 |
| CoV Angular Acceleration Stand-to-Sit, *%* | 26.6 ± 4.3 | 33.1 ± 9.3 | 0.525 | 0.30 |
| CoV Angular Velocity Stand-to-Sit, *%* | 15.4 ± 2.0 | 27.6 ± 10.5 | 0.245 | 0.553 |
| *s* represents time in seconds; metrics with *p values <0.05* were considered significantly different; PD, Parkinson’s Disease and PSP, Progressive supranuclear palsy, CoV: Coefficient of Variation | | | | |

| **Supplementary Table 4. Correlation of TUG metrics with PSPR-Gait and mPSPRS-21 Scores** | | | | |
| --- | --- | --- | --- | --- |
|  | PSPRS-Gait Score | | mPSPRS-21 Score | |
|  | Correlation | p value | Correlation | p value |
| Sit-to-Stand Transition, s | 0.65 | 0.043 | 0.84 | 0.005 |
| TUG Duration, s | 0.72 | 0.018 | 0.64 | 0.064 |
| Mid Turn Duration, *s* | 0.67 | 0.033 | 0.62 | 0.074 |
| Walk Towards, s | 0.51 | 0.13 | 0.58 | 0.10 |
| Last Turn Duration, *s* | 0.55 | 0.10 | 0.57 | 0.11 |
| Stand-to-Sit Duration, *s* | 0.15 | 0.68 | 0.24 | 0.53 |
| Walk Return, s | 0.43 | 0.215 | 0.23 | 0.54 |
| AP sway during Turning, *deg* | 0.224 | 0.533 | -0.25 | 0.486 |
| ML sway during Turning, *deg* | 0.031 | 0.932 | 0.03 | 0.933 |
| Pelvis sway velocity during Turning, *deg/s* | -0.474 | 0.167 | -0.482 | 0.159 |
| *s* represents time in seconds; *AP* represents anterior-posterior, *ML* represents medial-lateral; mobility metrics with *p values <0.05* were considered significantly different; PD, Parkinson’s | | | | |
